# Structural plasticity of the N-terminal capping helix of the TPR domain of kinesin light chain

**DOI:** 10.1371/journal.pone.0186354

**Published:** 2017-10-16

**Authors:** The Quyen Nguyen, Mélanie Chenon, Fernando Vilela, Christophe Velours, Magali Aumont-Nicaise, Jessica Andreani, Paloma F. Varela, Paola Llinas, Julie Ménétrey

**Affiliations:** 1 Laboratoire d'Enzymologie et Biochimie Structurales (LEBS), CNRS, Université Paris-Sud, 1 avenue de la Terrasse, Gif-sur-Yvette, France; 2 Institute for Integrative Biology of the Cell (I2BC), CEA, CNRS, Univ. Paris-Sud, Université Paris-Saclay, Gif-sur-Yvette cedex, France; Russian Academy of Medical Sciences, RUSSIAN FEDERATION

## Abstract

Kinesin1 plays a major role in neuronal transport by recruiting many different cargos through its kinesin light chain (KLC). Various structurally unrelated cargos interact with the conserved tetratricopeptide repeat (TPR) domain of KLC. The N-terminal capping helix of the TPR domain exhibits an atypical sequence and structural features that may contribute to the versatility of the TPR domain to bind different cargos. We determined crystal structures of the TPR domain of both KLC1 and KLC2 encompassing the N-terminal capping helix and show that this helix exhibits two distinct and defined orientations relative to the rest of the TPR domain. Such a difference in orientation gives rise, at the N-terminal part of the groove, to the formation of one hydrophobic pocket, as well as to electrostatic variations at the groove surface. We present a comprehensive structural analysis of available KLC1/2-TPR domain structures that highlights that ligand binding into the groove can be specific of one or the other N-terminal capping helix orientations. Further, structural analysis reveals that the N-terminal capping helix is always involved in crystal packing contacts, especially in a TPR1:TPR1’ contact which highlights its propensity to be a protein–protein interaction site. Together, these results underline that the structural plasticity of the N-terminal capping helix might represent a structural determinant for TPR domain structural versatility in cargo binding.

## Introduction

Kinesins are a superfamily of molecular motor proteins that move along microtubules powered by ATP hydrolysis energy [1]. The active movement of kinesins supports several cellular functions including cell division and transport of cellular cargos [2]. Defects of kinesin functions are involved in various pathologies, including cancer and nervous system, metabolic and cilia diseases [2–4]. Kinesin1 (also known as conventional kinesin or Kif5) plays a major role in neuronal transport by recruiting many different cargos such as organelles, vesicles, mRNA/proteins complexes and protein assemblies [5,6]. Accumulating evidence suggest a key role for kinesin1 in several neurological disorders including Alzheimer’s disease [7]. Kinesin1 functions as a hetero-tetramer composed of a dimer of kinesin heavy chains (KHC) bound to two kinesin light chains (KLC) [8]. KHC consists of three regions: a N-terminal globular motor domain (head) that contains the ATP and microtubule binding sites, a central elongated coiled-coil (stalk) responsible for dimerization, and a C-terminal unstructured region (tail) that regulates motor motility and recruits cargos. KLC is also composed of three regions: a N-terminal Heptad Repeat (HR) region that binds to the KHC stalk, a TPR (Tetratrico Peptide Repeat) domain involved in cargo recruitment, and a variable C-terminal region.

While only one KLC-like isoform has been found in invertebrates, four KLC isoforms (KLC1-4) have been identified in vertebrates. KLC1/2 isoforms bind several proteins that are associated with axonal transport and neurodegeneration, such as the structurally unrelated JIP1/2 and JIP3/4 (JNK-interacting proteins 1/2 and 3/4) cargos [9–12], as well as the growing family of W-acidic motif (tryptophan residue flanked by acidic residues) cargos including Huntingtin-Associated Protein-1 (HAP1), the type I transmembrane Calsyntenin-1/Alcadein α (CSTN1/Alcα) proteins or the lysosome adaptor SKIP (SifA-Kinesin Interacting Protein) [13–17]. Interestingly, among the different mechanisms that regulate kinesin1 cargo binding, one is KLC auto-inhibition [18]. Within the flexible linker between the HR region and the TPR domain of KLC there is a highly conserved Leucine-Phenylalanine-Proline (LFP) motif flanked by acidic residues that folds back on the TPR domain partly occluding the cargo binding site. However, while auto-inhibition by the LFP motif reduces KLC affinity for WD-motif cargo SKIP, it only marginally reduces affinity for JIP1 [18], suggesting that other mechanisms may regulate cargo binding to the KLC-TPR domain.

The basic function of TPR domains is to mediate protein–protein interactions and this can be achieved in a variety of ways [19]. TPR domains are present in a wide range of proteins consisting of several TPR motifs in tandem (from 3 to 16 repeats) [19]. Each motif repeat involves two antiparallel α helices (A and B) which stack together, in a parallel array relative to other motifs, to form an extended molecule with an overall right-handed super-helical architecture. The domain adopts a cradle-shape with helices A of each repeat lining the concave face (or groove) and helices B lining the convex face. A standard “nPR” nomenclature system has been proposed for the TPR family proteins based on variable-length TPR-motifs, where n represents the number of residues in a single repeat [20]. Accordingly, the canonical 34-residue TPR motif is referred to as 34PR motif, and the longer 42-residue motifs, as 42PR motif. The TPR domain of KLC1/2 belongs to the 42PR motif family and consists of six TPR motifs (TPR1-6) with a non-TPR region of 40 residues inserted between TPR5 and TPR6 motifs. 3D structures of KLC1/2 showed that the TPR domain adopts a classical TPR fold consisting of 12 helices with the partially flexible non-TPR region extruding from the convex side at the C-terminal part of the TPR domain [18,21,22].

Crystal structure of KLC2-TPR bound to the natural WD-motif peptide of SKIP revealed for the first time details of the WD-motif cargo binding mode [22]. SKIP-WD peptide binds inside the positively charged groove of KLC2-TPR inducing an overall rigid-jaw (closure) movement of the TPR domain that creates a hydrophobic pocket at the interface of TPR2 and TPR3 motifs [22]. The tryptophan residue from the WD motif plugs into the pocket, and this interaction is critical for SKIP recruitment by KLC2 [22]. A second, and distinct, hydrophobic pocket was identified at the interface of TPR1 and TPR2 motifs in the structure of KLC1-TPR [21] filled by a phenylalanine residue coming from the unnatural His-tag linker. Such an interaction, as the one observed in KLC2-TPR bound to SKIP-WD peptide, might also represent a structural determinant for cargo recognition and binding. Because this latter pocket is formed by residues from the N-terminal capping helix of the TPR domain, we aim to examine, based on structural data, its putative role in cargo recognition and binding.

Here, we report two crystal structures of the TPR domain of both KLC1 and KLC2 encompassing the N-terminal capping A1 helix. These structural data highlight, for the first time, the structural plasticity of this helix which can populate two distinct and defined orientations relative to the rest of the TPR domain. The reorientation of the N-terminal capping helix leads to the formation of one hydrophobic pocket into the groove at the TPR1-TPR2 interface that might be a recognition site for cargos. Based on the available crystal structures of the TPR domain of KLC1/2, we examine the orientation of the N-terminal capping helix according to the ligand-bound form, as well as to the crystal packing contacts.

## Materials and methods

### Gene constructs, protein expression and purification

cDNAs encoding the mouse KLC2-TPR^[A1-B6]^ (residues 190–484) fragment was cloned into the pET28a plasmid in Nde1/EcoR1 restriction sites, while the human KLC1-TPR^[A1-B5]^ (residues 185–418) fragment was cloned in Nde1/BamH1 restriction sites. KLC fragments were produced in *Escherichia coli* BL21-Gold(DE3) as N-terminus His-tag fusion protein. Cells were collected after induction with 0.3 mM IPTG overnight at 20°C. Frozen bacteria were suspended in 25 mM Hepes pH 7.0 containing 500 mM NaCl, 5% glycérol, 1mM DTT, 5 mM imidazole pH 7.0, 0.1% Triton, 1 mM PMSF, 5 μg /mL leupeptin, aprotenin and benzonase (1u/L, Sigma) and 0.7 mg/mL lysozyme. The lysate was incubated 1h at 4°C, disrupted by sonication and ultracentrifuged at 40 000 rpm for 40 min at 4°C. The soluble lysate was incubated at 4°C onto Ni^2+^-NTA (Amersham Biosciences) beads with 25 mM Hepes pH 7.0, 500 mM NaCl, 5% glycerol, 1 mM DTT and 5 mM imidazole pH 7.0 for 2 h and eluted with addition of 0.5 M imidazole. Then it was loaded on a Superdex 75 column equilibrated with 25 mM Hepes pH 7.0, 200 mM NaCl and 0.2 mM TCEP. KLC fragments were stored at -80°C at 3–7 mg ml^-1^.

### Protein crystallization, data collection and structure determination

Crystals of the KLC2-TPR^[A1-B6]^ fragment were obtained by the vapour diffusion method at 290 K in sitting drops using equal amounts of protein and reservoir solution. The crystallization solution consists of 0.5 M K2HPO4/Na2HPO4 pH 7.5, 12% PEG3350 and 1mM Sarcosine and the stock solution of the protein is at 5 mg ml^-1^. Crystals were transferred briefly to a cryo-protectant composed of reservoir solution supplemented with 25% glycerol and frozen in liquid nitrogen. Diffraction data were collected at 100 K on beamline PROXIMA-2 at the SOLEIL Synchrotron. Crystals diffract up to 3.4 Å and belong to the monoclinic space group C2 with three molecules in the asymmetric unit. X-ray data were integrated and scaled using XDS [23]. The structure was determined by molecular replacement with PHASER [24] using as a search model the TPR^[B1-B6]^ fragment from the mouse KLC2-TPR^[B1-B6]^ structure (PDB code 3CEQ). The A1 helix was then manually docked in the extra electron density using the human KLC1-TPR^[A1-B6]^ structure (PDB code 3NF1) as model. Refinement was then carried out using autoBuster [25] with the autoNCS option [26] and the graphical building was performed using COOT [27]. It should be noted that the 4 N-terminal residues (190–193) from KLC2-TPR^[A1-B6]^ fragment, as well as the 19 residue-stretch containing the His-tag and the thrombine cleavage site were not modeled due to electron density absence. Also, the 24 residues (421–444) from the non-TPR region between the TPR5 and TPR6, as well as the 5 last C-terminal residues are not modelled due to electron density absence. No disulfide bridge is observed between Cys441 (not visible in electron density) and Cys474 (visible in electron density). Atomic coordinates and structure factors have been deposited in the PDB (code 5OJF).

Crystals of the KLC1-TPR^[A1-B5]^ fragment were obtained by the vapour diffusion method at 290 K in sitting drops using equal amounts of protein and reservoir solution. The crystallization solution consists of 22% PEG400, 10% PEG1000 and 150 mM Phosphate pH 6.5 and the stock solution of the protein is at 7 mg ml^-1^. Crystals were frozen in liquid nitrogen. Diffraction data were collected at 100 K on beamline PROXIMA-1 at the SOLEIL Synchrotron. The crystals diffract up to 2.25 Å and belong to the hexagonal space group P6_2_22 with one molecule in the asymmetric unit. X-ray data were integrated and scaled using XDS [23]. The structure was determined by molecular replacement with PHASER [24] using as a search model the TPR^[A1-B4]^ from the KLC1-TPR^[A1-B6]^ structure (PDB code 3NF1). The fifth TPR repeat was then manually docked in the extra electron density using COOT [27]. Refinement was then carried out using Phenix [28] and the graphical building was performed using COOT [27]. It should be noted that the 24 N-terminal residues (185–208) from KLC1-TPR^[A1-B5]^, as well as the 21-residue stretch containing the His-tag and the thrombine cleavage site, were not modeled due to electron density absence. Atomic coordinates and structure factors have been deposited in the PDB (code 5OJ8).

For both structures, data collection and refinement statistics are presented in Table 1. *MolProbity* was used for model validation [29]. Figures were produced using Pymol [30].

**Table 1:** Data-collection and refinement statistics.

Fragment	KLC2-TPR^[A1-B6]^	KLC1-TPR^[A1-B5]^
PDB code	5OJF	5OJ8
**Data collection**		
Beamline	PX2, Soleil	PX1, Soleil
Wavelength (Å)	0.980097	0.978570
Space group	C2	P6_2_ 2 2
Unit-cell parameters		
a, b, c (Å)	97.62, 116.47, 108.11	69.53, 69.53, 199.73
α, β, γ (°)	90.00, 99.51, 90.00	90.00, 90.00, 120.00
Resolution (Å)	48.14–3.40 (3.59–3.40)	44.66–2.25 (2.38–2.25)
Unique reflections	16593 (2566)	14301 (2201)
C/C1/2†	98.8 (63.7)	100.0 (42.7)
R-meas (%)	23.6 (116.8)	6.6 (152.4)
Mean I/σ(I)	4.55 (1.08)	21.25 (1.10)
Completeness (%)	99.2 (95.7)	98.6 (97.5)
Multiplicity	3.6 (3.9)	8.6 (5.7)
**Refinement**		
Resolution (Å)	20.00–3.40 (3.63–3.40)	34.25–2.25 (2.42–2.25)
No. of reflections	16409 (2984)	14294 (2733)
R-work (%)	23.2 (22.3)	20.4 (31.4)
R-free‡ (%)	26.4 (27.8)	24.0 (38.0)
No. of atoms	6255	1702
Mean *B* value (Å^2^)	127.8	67.1
R.m.s.d. from ideal		
Bond lengths (Å)	0.010	0.009
Bond angles (Deg)	1.190	0.930
Ramachandran plot ¶		
Favoured (%)	91.1	98.6
Allowed (%)	7.3	1.4

Values in parenthesis are for the highest resolution shells.

†*C/C1/2* = ###.

‡*R*_work_ = Σ_**hkl**_**||F**_**obs**_**|–k|F**_**calc**_**||/**Σ_**hkl**_**|F**_**obs**_**|** for 95% of the reflection data used in refinement. *F*_obs_ and *F*_calc_ are the observed and calculated structure-factor amplitudes, respectively. ‡*R*_free_ is the equivalent of *R*_work_ except that it was calculated for a randomly chosen 5% test set excluded from refinement.

¶ Ramachandran analysis was performed using *MolProbity* [29].

### Structure analysis

Crystal packing contact analysis and surface area calculation were performed using the interactive tool PDBePISA [31]. The root mean square deviation (rmsd) was calculated using the CCP4 suite [32]. Helix crossing angles and distances were calculated using helix_angles.py (R.L. Campbell, Queen University). To evaluate TPR domain closure and the A1 helix orientation, we fixed αA2 as the reference helix and calculated helix crossing angle and distance using A5 and A1 helices, respectively. These allow to take in account the absence of αA1, αA2 and TPR6 helices from some KLC structures (Table 2). Because B5 helix in the KLC1-TPR^[A1-B5]^ structure (this study) is slightly rotated, due to tight crystal contact, we decided to use A5 helix reference to evaluate TPR domain closure.

**Table 2:** Information from crystal structures of the TPR domain of KLC.

	Res. (Å)	Fragments	SG	Mol/ua	Ligand-bound	Ligand-type
**KLC1**						
5OJ8	2.25	[A1-B5]	P6_2_22	1	αB5’-sym	Unnatural
3NF1	2.8	[A1-B6]	P3_1_21	1	His-tag	Unnatural
**KLC2**						
5OJF	3.4	[A1-B6]	C2	3	-	-
5FJY	4.0	LFP-[A1-B6]	C2	3	LFP-motif	Natural
3CEQ	2.75	[B1-A6]	P2_1_2_1_2_1_	2	-	-
3EDT	2.7	[B1-A6]	P3_1_	4	-	-
3ZFW	2.9	*[B1-A6]	P2_1_2_1_2	2	SKIP-WD	Natural

* although, the construct includes the B1 helix, this latter is not modeled in the structure due to unambiguously interpreted electron density.

### Size-exclusion chromatography coupled to multi-angle laser light scattering

Protein samples were analysed by Size-Exclusion Chromatography (SEC) coupled with Multi-Angle Laser Light Scattering (MALLS). KLC1-TPR^[A1-B5]^ and KLC2-TPR^[A1-B6]^ were loaded on a Superdex 200 10/300 GL increase column (GE Healthcare) equilibrated respectively with 50mM Hepes pH 8.0, 80mM NaCl; 5% glycérol, 1mM DTT and 20mM Hepes pH 7.0, 300mM at a flow rate of 0.5 ml/min on a HPLC system (Shimadzu). Static light scattering was measured with a MiniDAWN TREOS (Wyatt Technology), protein concentration was determined using an Optilab T-rEX refractometer (Wyatt Technology) and dynamic light scattering was carried out with a WyattQELS (Wyatt Technology). The data were analysed using ASTRA® 6.1 software (Wyatt Technology). A 0.183 ml g^-1^ refractive index increment (dn/dc) was used to calculate protein concentrations and absolute molar masses.

## Results and discussion

### N-terminal capping helix of the TPR domain exhibits differences compared to other A helices

**Fig 1:**
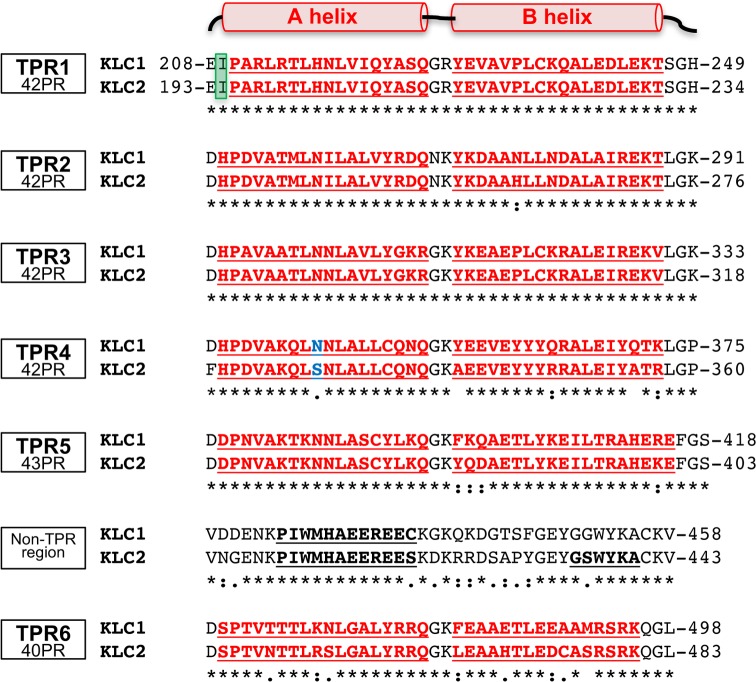
Structure-based sequence alignment of the TPR domain of KLC1 and KLC2. Sequence alignment is organized by TPR motif. Sequence accession number for human KLC1 is AAH08881.1a, and for mouse KLC2 is NP_032477.2. Helices A and B from each TPR motif are underlined and indicated in red. Helices in the non-TPR region are also indicated in bold. Position 2 of the TPR1 motif that differs from other TPR motifs is highlighted in green. Sequence difference between KLC1 and KLC2 that discriminate JIP1 cargo binding is colored in blue.

As mentioned before, the TPR domain of KLC belongs to the 42PR family with helices A and B longer by 3 and 4 residues, respectively, compared to the canonical 34PR family [20]. However, sequence analysis of the TPR domain of KLC1 and KLC2 isoforms reveals that TPR1/2/3/4 consist of 42 residues, while TPR5 is longer with 43 residues and TPR6 shorter with 40 residues (Fig 1). Such difference in the TPR motif length involves B5 helix is longer by 1 residue and B6 helix is shorter by 2 residues compared to B1/2/3/4 helices. Overall, KLC1 and KLC2 share 89.7% of their sequence identity within their TPR domain. But sequence alignment shows that the N-terminal part of the TPR domain of KLC1 and KLC2 is more conserved than the C-terminal part. Indeed, TPR1/2/3 are highly conserved with pairwise sequence identity (PSI) of 100, 97.6 and 100%, respectively, while TPR4/5/6 are less conserved with PSI about 85.7, 90.7 and 80.0%, respectively. Furthermore, the A helices are more conserved than B helices among the six TPR motifs with A helices of TPR1/2/3 and TPR5 being 100% identical between KLC1 and KLC2. However, despite a high sequence identity conservation between KLC1 and KLC2, especially in the TPR domain groove, a single difference in the sequence can change the specificity for ligand binding. For instance, the kinesin1 adaptor protein, JIP1, discriminates between KLC1 and KLC2 due to one sequence difference on the A4 helix (Fig 1; Asn343 in KLC1 that is replaced by Ser328 in KLC2) [21].

Structural analysis reveals that, despite the 42PR motif length of TPR1, the N-terminal capping A1 helix is shortened by 1 residue at its N-terminus compared to other A helices of the TPR domain. This is due to a sequence difference at position 2 of the 42PR motif (Fig 1). In TPR2/3/4/5/6, the position 2 is occupied by a polar residue, mainly a histidine which stabilizes the first helix turn by making a hydrogen bond with the helix backbone, while in TPR1, position 2 is occupied by a hydrophobic residue (KLC1-Ile209 and KLC2-Ile194) than can no longer make this hydrogen bond. Consequently, the strictly conserved proline residue at position 3 is less constrained and exhibits a 180° difference in its Ψ torsion angle. Thus, while the N-terminal part of the TPR domain is highly conserved, especially the A helices of KLC, the A1 helix exhibits one sequence difference that leads to structural differences. These differences should be taken into account to fully understand the versatility of the TPR domain of KLC for protein-protein interactions.

### Crystal structure of the unbound KLC2-TPR allows examination of its N-terminal capping helix upon LFP-motif binding

**Fig 2:**
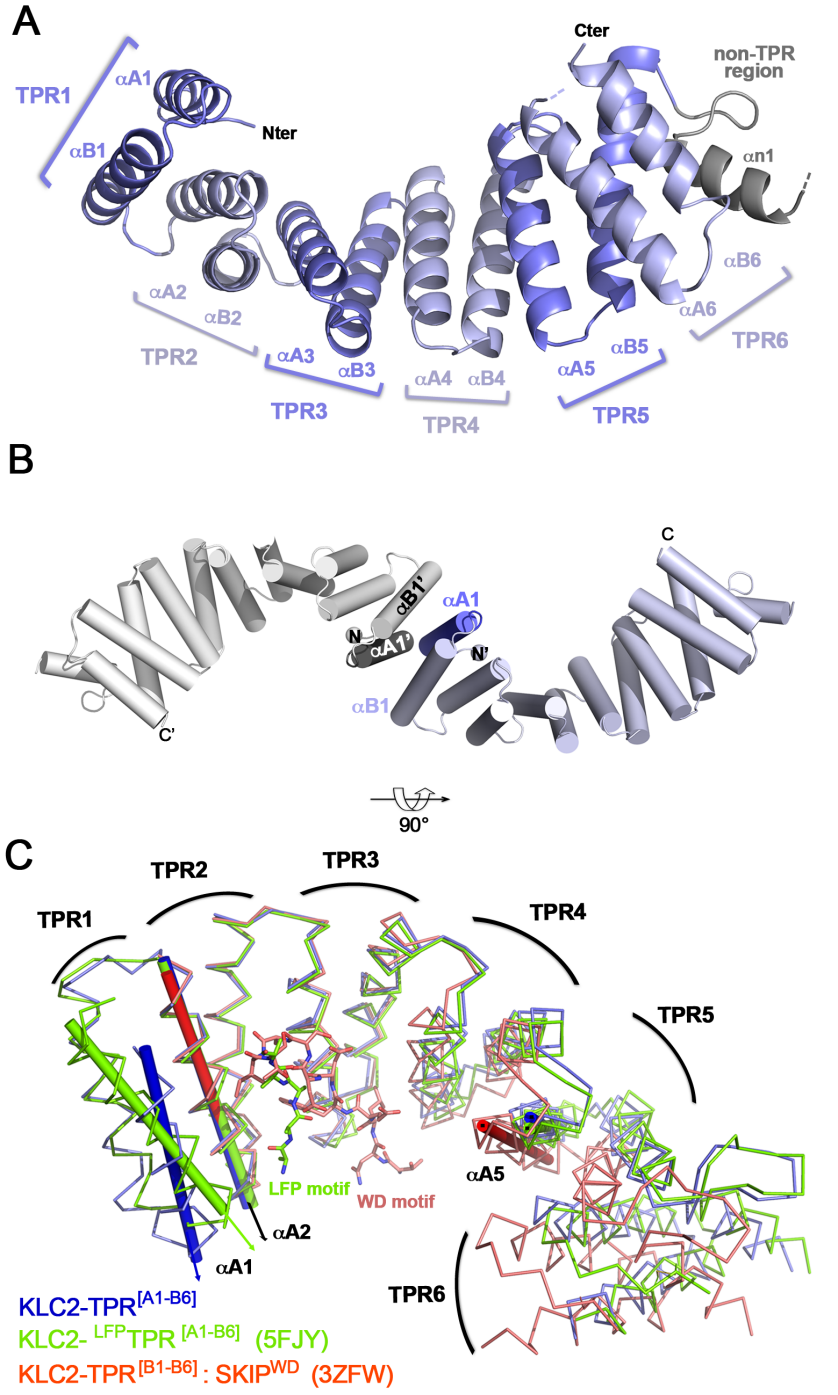
Crystal structure of the complete TPR domain of KLC2. (A) 3D structure of KLC2-TPR^[A1-B6]^. (B) A cartoon representation of the TPR1:TPR1’ crystal packing contact. The A1 helix is indicated in dark blue. The crystal contact molecule is coloured in white and labelled with an apostrophe. (C) Superposition of KLC2-TPR^[A1-B6]^ (blue), KLC2-^LFP^TPR^[A1-B6]^ (5FJY, green) and KLC2-TPR^[B1-B6]^:SKIP^WD^ (3ZFW, red) structures. The TPR domain superposition is done on the TPR2 motif. Axes of A1, A2 and A5 helices are indicated with thin cylindrical tubes. The TPR domain curvature and the αA1 orientation can be observed by comparing αA5 and αA1 axis to the reference αA2 axis.

In order to analyse the structural impact of LFP-motif binding on the TPR domain of KLC, especially at the TPR1 motif, we crystallized an unbound complete TPR domain of KLC2 and compared it to the previously solved KLC2-TPR bound to LFP-motif [18]. The crystal structure of our KLC2-TPR fragment encompassing TPR motifs 1 to 6 (namely hereafter KLC2-TPR^[A1-B6]^; residues 190–484) was determined at 3.4 Å resolution with three molecules in the asymmetric unit (PDB code 5OJF; data collection and refinement statistics are listed in Table 1). This fragment of KLC2 lacks the flexible linker encompassing the LFP motif. The structure of KLC2-TPR^[A1-B6]^ adopts the common KLC-TPR structural fold composed of 12 α-helices (namely A1 to B6 helices; Fig 2A). The non-TPR region of 40 residues connecting the TPR5 motif to the TPR6 motif is made up of an α-helix (αn1; residue 410–420), followed by a disordered region (421–444) that is not modeled in the crystal structure due to the absence of electron density. Interestingly, the three KLC2-TPR^[A1-B6]^ molecules make the same crystal packing contact with a 2-fold axis symmetrical counterpart leading to TPR1:TPR1’ interactions. The two anti-parallel A1/B1 helices from each molecule are roughly perpendicular to each other (Fig 2B) and bury a total surface area of 1533 Å^2^ involving numerous hydrophobic residues. Because of this crystal contact, we evaluate the oligomeric state of KLC2-TPR^[A1-B6]^ in solution. SEC-MALLS experiment reveals that KLC2-TPR^[A1-B6]^ is a monomer in solution with a weight-averaged mass of 36.5 ± 1.0 kDa (S1A Fig). Thus, this TPR1:TPR1’ contact alone is not sufficient to stabilize such a dimer in solution.

Structural comparison of KLC2 unbound (KLC2-TPR^[A1-B6]^; this study) and bound to its LFP-motif (KLC2-^LFP^TPR^[A1-B6]^; PDB code 5FJY, Table 2, [18]) shows no overall closure of the TPR domain upon LFP-motif binding, in contrast to what has been observed upon SKIP-WD motif peptide binding (KLC2-TPR^[B1-B6]^:SKIP^WD^; PDB code 3ZFW; [22]) (Fig 2C). Indeed, the average distances between A2 and A5 helices are 31.5 Å and 31.3 Å in KLC2-TPR^[A1-B6]^ and KLC2-^LFP^TPR^[A1-B6]^ (5FJY) structures, respectively, while the distance in KLC2-TPR^[B1-B6]^:SKIP^WD^ (3ZFW) structure decreases to 28.0 Å (calculated on Cα atoms; Table 3). Also, secondary structure matching of both KLC2-TPR^[A1-B6]^ (this study) and KLC2-^LFP^TPR^[A1-B6]^ (5FJY) structures against KLC2-TPR^[B1-B6]^:SKIP^WD^ (3ZFW) structure gives a rmsd of 1.8 Å and 2.0 Å (aligned on Cα atoms of [A2-B6] fragment; 219 residues), respectively. This value decreases to 0.8 Å when matching is done between KLC2-TPR^[A1-B6]^ (this study) and KLC2-^LFP^TPR^[A1-B6]^ (5FJY) structures for the same residues range. When secondary matching is performed on the complete TPR domain (aligned on Cα atoms of [A1-B6] fragment; 262 residues), encompassing the TPR1 motif, the rmsd increases to 2.5 Å between KLC2-TPR^[A1-B6]^ (this study) and KLC2-^LFP^TPR^[A1-B6]^ (5FJY) structures. This variance highlights structural differences at the TPR1 motif. Structural comparison reveals that both TPR1 helices change their orientation relative to the rest of the TPR domain, especially the N-terminal capping A1 helix that is in proximity to the LFP-motif peptide (Fig 2C). Indeed, the crossing angle between αA1 and αA2 axis is 7.0° in KLC2-TPR^[A1-B6]^ structure (this study), while it is 21.4° for KLC2-^LFP^TPR^[A1-B6]^ (5FJY) structure (calculated on Cα atoms, Table 4 and Fig 2C). Furthermore, A1 helix in KLC2-TPR^[A1-B6]^ (this study) structure shifts along its axis by one helix turn towards its C-terminus relative to that of KLC2-^LFP^TPR^[A1-B6]^ (5FJY) structure (Fig 2C). It is also noteworthy that the orientation of A1 and B1 helices observed in KLC2-^LFP^TPR^[A1-B6]^ (5FJY) structure cannot accommodate in the KLC2-TPR^[A1-B6]^ (this study) crystal packing without generating drastic helix clashes at the TPR1:TPR1’ contact. Finally, because of the crystal packing contacts at the TPR1 motif, we cannot elucidate what component of the crystal packing contacts or the binding of the LFP-motif triggers the A1 helix reorientation observed in KLC2-TPR^[A1-B6]^ (this study) structure.

**Table 3:** Helix crossing measurements for TPR domain closure.

		(αA2 - αA5)		TPR	αA2/αA3
	Fragments	Angle (°)	Dist. (Å)	Conformation	pocket
**KLC1**					
5OJ8_A	[A1-B5]	55.0	26.8	CLOSE	Yes
3NF1_A	[A1-B6]	49.8	29.9	Intermediate	No
**KLC2**					
5OJF_B	[A1-B6]	62.9	31.5	OPEN	No
5FJY_A	LFP-[A1-B6]	66.2	31.3	OPEN	No
3CEQ_A	*[B1-A6]	65.8	32.8	OPEN	No
3EDT_H	*[B1-A6]	67.9	31.2	OPEN	No
3ZFW_A	*[B1-A6]	54.3	28.0	CLOSE	Yes

*the A1 helix is absent from the TPR fragment crystallized, thus A1 helix crossing measurements cannot be calculated.

**Table 4:** Helix crossing measurements for A1 helix movement.

		(αA1 - αA2)			αA1	αA1/αA2
	Fragments	Angle (°)	Dist. (Å)	ASA (Å^2^)	orientation	pocket
**KLC1**						
5OJ8_A	[A1-B5]	6.3	9.5	244	IN	No
3NF1_A	[A1-B6]	19.9	10.8	145	OUT	Yes
**KLC2**						
5OJF_B	[A1-B6]	7.0	10.0	NC	IN	No
5FJY_A	LFP-[A1-B6]	21.4	12.0	NC	OUT	Yes

NC: Not Calculated

### A new crystal form of KLC1-TPR confirms a distinct N-terminal capping helix orientation

**Fig 3:**
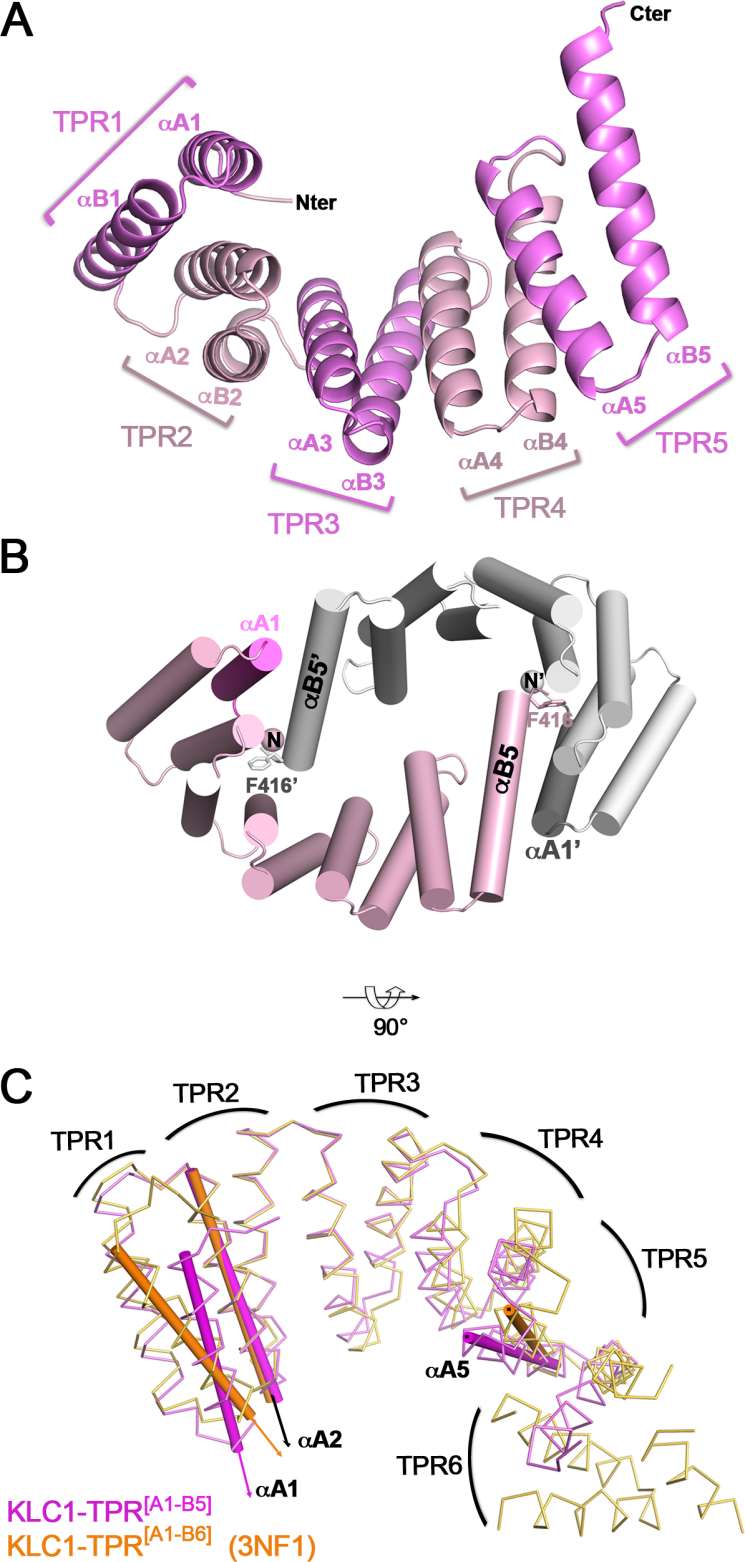
Crystal structure of a C-terminal truncated TPR domain of KLC1. (A) 3D structure of KLC1-TPR^[A1-B5]^. (B) Cartoon representation of the αA1:αB5’-sym crystal packing contact. The A1 helix is indicated in magenta. The crystal contact molecule is coloured in white and a labelled with an apostrophe. The Phe416 residue is shown in sticks. (C) Superposition of KLC1-TPR^[A1-B5]^ (this study, pink) and KLC1-TPR^[A1-B6]^ (3NF1, orange; the N-terminal His-tag is removed for clarity) structures. TPR domain superposition is done on the TPR2 motif. The axes of A1, A2 and A5 helices are indicated with thin cylindrical tubes. The TPR domain curvature and the αA1 orientation can be observed by comparing the αA5 and αA1 axes to the reference αA2 axis.

The TPR domain of KLC1 (KLC1-TPR^[A1-B6]^, residues 205–497, [21]) was previously crystallized in presence of its N-terminal His-tag. The 3D structure (PDB code 3NF1, Table 2) shows that the His-tag linker folds back and binds into the groove, interacting extensively with A1, A2 and A3 helices. In order to visualise the structure of KLC1-TPR domain without this unnatural ligand bound, we created a new fragment of KLC1 that differs in its N-terminal His-tag linker sequence. Due to difficulties in crystallization, this fragment lacks the non-TPR region and the TPR6 motif. Thus, we determined the 3D structure of human KLC1 encompassing the TPR motifs 1 to 5 (namely hereafter KLC1-TPR^[A1-B5]^; residues 185–418) at 2.25 Å resolution with one molecule in the asymmetric unit (PDB code 5OJ8; data collection and refinement statistics are listed in Table 1). The structure of KLC1-TPR^[A1-B5]^ is composed of 10 α-helices (namely A1 to B5 helices) adopting the common KLC-TPR structural motif (Fig 3A). The region upstream the TPR domain encompassing the His-tag linker has no electron density and was not modeled. So, and in contrast to what is observed in the KLC1-TPR^[A1-B6]^ structure (3NF1), in the KLC1-TPR^[A1-B5]^ structure, the His-tag linker does not fold back into the groove. However, in the crystal, the KLC1-TPR^[A1-B5]^ molecule makes a tight interaction with one of its symmetrical counterparts. Through a 2-fold axis, both C-terminal helices (αB5) bind into the N-terminal part of the groove of the related molecule (Fig 3B), covering a total buried surface area of 3196 Å^2^. Worthy of note, such a dimeric assembly should not be observed with a complete TPR domain because of steric clashes between the N-terminal part of the TPR domain and the non-TPR region of the related molecule. The N-terminal part of the TPR domain consisting of A1, A2, B2 and A3 helices makes extensive hydrophilic and hydrophobic interactions with the B5’ helix (K405’-F416’), as well as with the last two extreme C-terminus residues (G417’-S418’) from the symmetrical molecule (hereafter denoted by an apostrophe). Among these, there are 7 hydrogen bonds, as well as 2 salt bridges between αA2-Arg266 and αB5’-Glu415’, and between α2B-Arg285 and αB5’^COOH^-Ser418’ (S2A Fig). The phenylalanine residue, Phe416’, located at the end of αB5’, is fully buried within a hydrophobic pocket formed at the interface of αA2 and αA3 (αA2/αA3 pocket) and composed of residues Asn259, Ala262, Leu263, Arg266 from αA2, Ala274 and Leu278 from αB2, Asn302, Val305 and Leu306 from αA3 (S2B Fig). Interestingly, the last 4 residues (E415’-S418’) of KLC1-TPR^[A1-B5]^ symmetry lie into the groove superposing with the N-terminal part (W207-I212) of the WD-motif of SKIP from KLC2-TPR^[B1-B6]^:SKIP^WD^ structure (3ZFW). Specially, the side-chain of Phe416’ occupies the same hydrophobic αA2/αA3 pocket as the Trp207 from SKIP-WD motif (S2C Fig). Because of this tight crystal packing, we investigate the oligomeric state of KLC1-TPR^[A1-B5]^ in solution using SEC-MALLS. A single peak was observed with a weight-averaged mass of 29.4 ± 0.2 kDa which indicates that the KLC1-TPR^[A1-B5]^ is a monomer in solution (S1B Fig). Thus, despite the extensive surface area buried and interaction network, this KLC1-TPR^[A1-B5]^ crystal dimer is not observed in solution.

Comparing the KLC1-TPR^[A1-B5]^ and KLC1-TPR^[A1-B6]^ structures (3NF1; [21], Table 2) reveals two main differences (Fig 3C). Firstly, the TPR domain of KLC1-TPR^[A1-B5]^ exhibits a closure with respect to that of KLC1-TPR^[A1-B6]^ (3NF1). Indeed, the distance between αA2 and αA5 axes is 26.8 Å for KLC1-TPR^[A1-B5]^, while that of KLC1-TPR^[A1-B6]^ (3NF1) increases to 29.9 Å (Table 3). As a reminder, the distance between αA2 and αA5 axis in KLC2-TPR^[B1-B6]^:SKIP^WD^ structure is 28.0 Å. Therefore, KLC1-TPR^[A1-B5]^ is also closer than KLC2-TPR^[B1-B6]^ bound to SKIP-WD motif (3ZFW). The TPR domain closure of KLC1-TPR^[A1-B5]^ is certainly the consequence of the tight crystal contact that drives the C-terminus of the B5’ helix to lie into the groove with Phe416’ occupying the αA2/αA3 pocket. Secondly, the A1 helix adopts a distinct orientation in KLC1-TPR^[A1-B5]^ structure compared to that in KLC1-TPR^[A1-B6]^ (3NF1) structure with an (αA1, αA2) crossing angle of 6.3° and 19.9°, respectively (Table 4). In addition, A1 helix in KLC1-TPR^[A1-B5]^ (this study) structure shifts along its axis by one helix turn towards its C-terminus relative to that of KLC1-TPR^[A1-B6]^ (3NF1) (Fig 3D). Finally, because of the tight crystal packing contact at the TPR1, we cannot conclude whether the αA1 reorientation observed in KLC1-TPR^[A1-B5]^ (this study) structure is due to the absence of the His-tag linker interactions into the groove or to the crystal packing.

### The N-terminal capping A1 helix exhibits two distinct orientations

**Fig 4:**
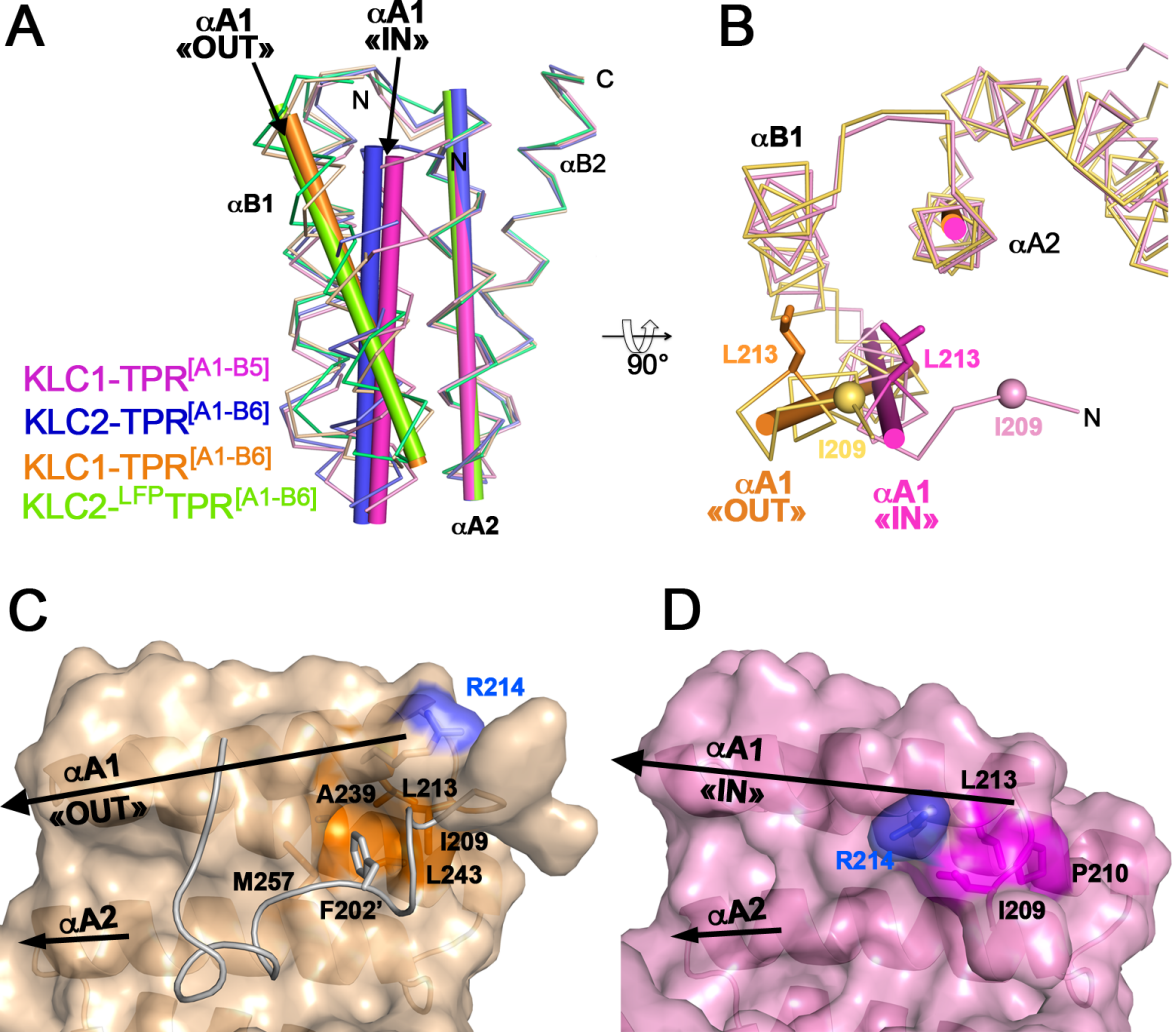
The N-terminal capping A1 helix exhibits two distinct positions. (A) Superposition of KLC1-TPR^[A1-B5]^ (this study; pink), KLC1-TPR^[A1-B6]^ (3NF1, orange), KLC2-TPR^[A1-B6]^ (this study, blue) and KLC2-^LFP^TPR^[A1-B6]^ (5FJY, green). TPR domain superposition is done on the TPR2 motif. (B) Orthogonal view of the superposition between KLC1-TPR^[A1-B5]^ (this study; pink), and KLC1-TPR^[A1-B6]^ (3NF1, orange). Residue L213 is shown in sticks. (C-D) Surface representation of the TPR1-TPR2 motifs of KLC1-TPR^[A1-B6]^ (3NF1, orange) and KLC1-TPR^[A1-B5]^ (this study, pink) with the same orientation. This view faces the groove. Residues forming the αA1/αA2 hydrophobic pocket are colored in orange. The His-tag linker is shown in ribbon (grey) and its Phe202* is indicated in sticks. The positive charge group of Arg214 is shown in blue. Both A1 and A2 helices are highlighted by black arrows.

To date, four structures of KLC-TPR fragments encompassing the N-terminal capping A1 helix have been determined (Table 2): (i) KLC1-TPR^[A1-B5]^ (this study); (ii) KLC1-TPR^[A1-B6]^ (3NF1; [21]); (iii) KLC2-TPR^[A1-B6]^ (this study) and (iv) KLC2-^LFP^TPR^[B1-B6]^ (5FJY, [18]). Their structural comparison highlights two distinct orientations for the N-terminal capping A1 helix. First, in KLC1-TPR^[A1-B6]^ (3NF1) and KLC2-^LFP^TPR^[B1-B6]^ (5FJY) structures, the A1 helix shares the same orientation, packing against A2 helix with (αA1, αA2) angles of 19.9° and 21.4°, respectively (Table 4 and Fig 4A). Interestingly, these (αA1, αA2) angles are typical values for αA_i_-αB_i_-αA_i+1_ arrangement in TPR motif tandem [20]. This αA1 orientation will be referred to as “OUT” hereafter. Second, in KLC1-TPR^[A1-B5]^ and KLC2-TPR^[A1-B6]^ structures (this study), the A1 helix shares the same orientation, packing against A2 helix with (αA1, αA2) angles of 6.3° and 7.0 Å, respectively (Table 4 and Fig 4A). This αA1 orientation will be referred to as “IN” hereafter. Thus, these two distinct αA1 orientations differ by a rotation of about 14°, as well as a translation along its own axis by nearly one helix turn (5.0 Å at Ser225-Cα). Altogether, available structural data highlight two well-ordered αA1 orientations, which have been observed each twice in different crystal forms and ligand-bound forms supporting that the N-terminal capping A1 helix exhibits a structural plasticity.

Details of the interactions of the two A1 helix orientations related to the rest of the TPR domain are conserved between KLC1 and KLC2, which are strictly identical in sequence along the first 1.5 TPR motifs (A1, B1, and A2 helices; Fig 1). These interactions are described below based on KLC1-TPR^[A1-B6]^ (3NF1) and KLC1-TPR^[A1-B5]^ (this study) structures which have been determined at a higher resolution than the KLC2 structures (Table 2). In KLC1-TPR^[A1-B6]^ (3NF1) structure, the “OUT” A1 helix, makes few contacts with A2 helix (interface area of 145 Å^2^, Table 4), but largely packs against B1 helix with an interface area of 398 Å^2^. By contrast, in KLC1-TPR^[A1-B5]^ structure (this study), the “IN” A1 helix is brought closer to the A2 helix and thus packs between both B1/A2 helices. The interface area of A1 helix with A2 helix increases to 244 Å^2^ (Table 4), while that with B1 helix decreases to 264 Å^2^. As a consequence, in the KLC1-TPR^[A1-B6]^ (3NF1, “OUT” αA1) structure, Leu213 from the A1 helix is partially exposed, whereas in KLC1-TPR^[A1-B5]^ (this study, “IN” αA1) structure, it is fully buried at the αB1/αA2 interface; Leu213 shifts by 6.5 Å between the two αA1 orientations (Fig 4B). Worthy of note, the conserved hydrophobic Ile209 at position 2 of the TPR1 motif (Fig 1) contributes to the αB1/αA2 hydrophobic interface in both αA1 orientations. Whereas in other TPR motifs, position 2 is occupied by polar residues (His, Asp and Ser) that are exposed at the edge of the TPR groove due to the Ψ torsion angle flip of the conserved proline residue at position 3.

Does the structural plasticity of the A1 helix affect the TPR domain groove surface properties? In KLC1-TPR^[A1-B6]^ structure (3NF1, “OUT” αA1), a hydrophobic pocket is found at the N-terminus of αA1/αA2 interface (Fig 4C). This hydrophobic pocket is formed by residues Ile209 (position 2 of the TPR1 motif, Fig 1) and Leu213 from αA1, as well as Ala239 and Leu243 from αB1 and Met257 from αA2. Interestingly, this pocket is occupied by a phenylalanine (Phe202*) residue from the His-tag linker present in the KLC1 fragment crystallized (Fig 4C) which indicates the structural ability of such interaction. In KLC1-TPR^[A1-B5]^ structure (this study, “IN” αA1), no hydrophobic pocket is found at the αA1/αA2 interface and residues Pro210 and Leu213 from the αA1, as well as Ile209, occupy this space (Fig 4D). The A1 helix reorientation modifies also the electrostatic potential surface of the TPR domain groove. In KLC1-TPR^[A1-B6]^ structure (3NF1, “OUT” αA1), the positive charged group of Arg214 locates at the edge of the groove (Fig 4C), while in KLC1-TPR^[A1-B5]^ structure (this study, “IN” αA1), it exposes inside the groove (Fig 4D). The rearrangement of the A1 helix between the “IN” and the “OUT” orientation gives rise to significant structural differences in the groove surface of the TPR domain. Therefore, the structural plasticity of the N-terminal capping A1 helix can represent a structural determinant for cargo specificity.

### Relationship between the N-terminal capping helix orientation and the ligand-bound form

**Fig 5:**
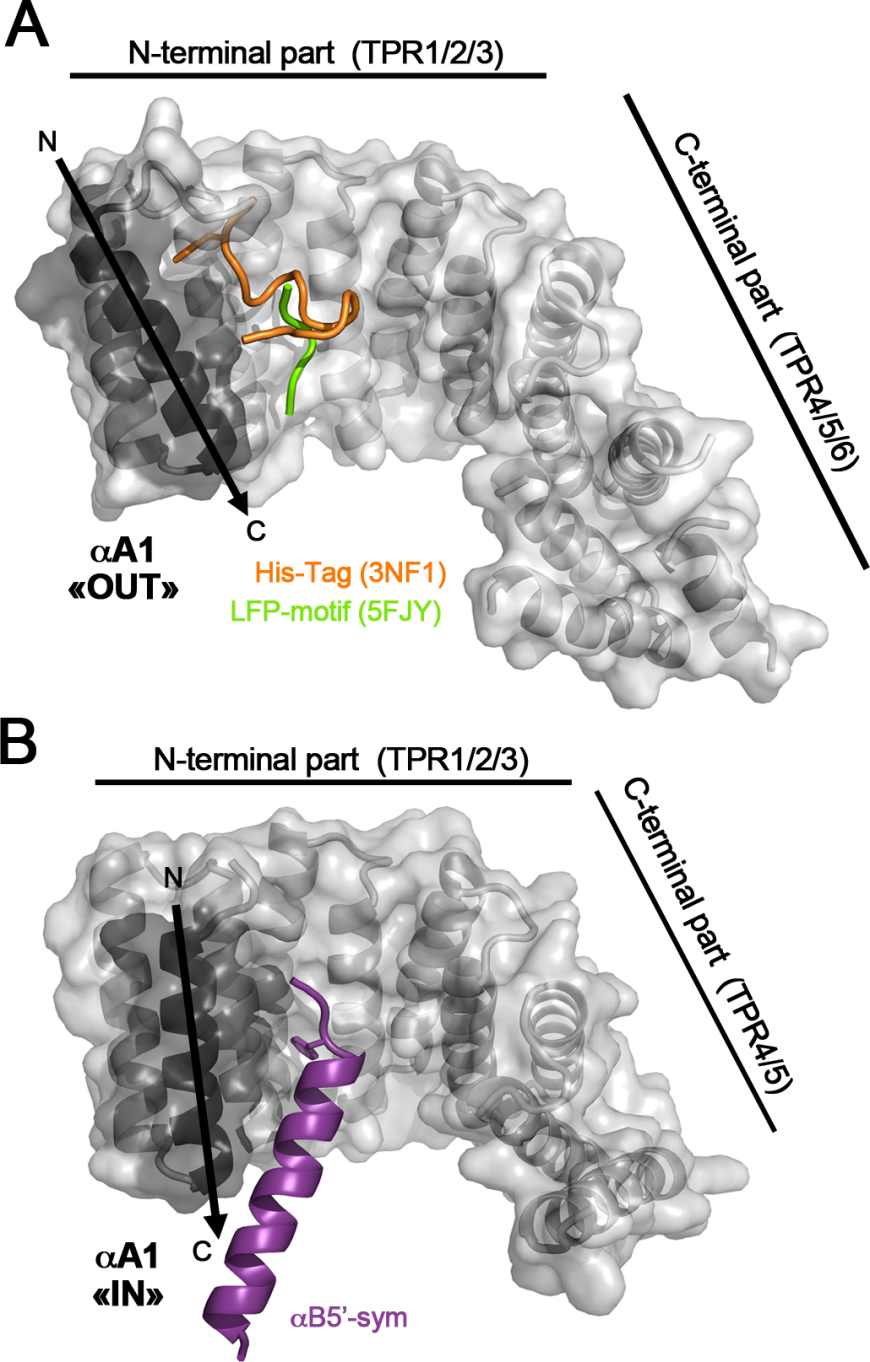
Relationship between the N-terminal capping A1 helix position and the TPR groove content. (A) Superposition of the unnatural His-tag fusion of KLC1-TPR^[A1-B6]^ (3NF1, orange) and the natural LFP-motif from KLC2-^LFP^TPR^[A1-B6]^ (5FJY, green). TPR domain superposition is done on the TPR2 motif. Only the KLC1-TPR[A1-B6] structure (3NF1) is shown for clarity in white cartoon surrounded by a transparent surface representation. The “OUT” A1 helix is indicated in dark grey. The Phe202* from the His-tag linker (3NF1, orange) is shown in sticks. (B) The KLC1-TPR^[A1-B5]^ (this study) contacting the B5’-sym helix (pink). KLC1-TPR^[A1-B5]^ is shown in white cartoon surrounded by a transparent surface representation. The “OUT” A1 helix is indicated in dark grey. The Phe416’ from the αB5’-sym (pink) is shown in sticks.

Among the four KLC1/2-TPR fragments encompassing the N-terminal capping A1 helix crystallized: (i) one is unbound (KLC2-TPR^[A1-B6]^, this study), (ii) one is bound to natural ligand (KLC2-^LFP^TPR^[A1-B6]^ bound to the internal LFP-motif, 5FJY, [18]), and (iii) two are bound to unnatural ligands (KLC1-TPR^[A1-B6]^ bound to its own His-tag linker (3NF1, [21]) and KLC1-TPR^[A1-B5]^ bound to the B5’ helix of a symmetry molecule (αB5’-sym; this study)). In order to analyze the relationship between the αA1 orientation and the ligand-bound form, we performed structural comparisons between these four KLC1/2-TPR forms. In the KLC2-TPR^[A1-B6]^ structure (this study) which is the unique unbound form, the A1 helix exhibits a “IN” orientation, while in the three ligand-bound forms, the A1 helix exhibits one or the other orientation (Fig 5). All these natural and unnatural ligands bind to the N-terminal part of the groove and partially superpose, but the details of their interaction with KLC differ (S3 Fig). In KLC2-^LFP^TPR^[A1-B6]^ structure (5FJY), the natural LFP-motif is close to the C-terminus of the A1 helix which exhibits an “OUT” orientation (Fig 5A). But due to the low resolution of this structure, it is not possible to determine if the A1 helix interacts directly with the LFP motif. Further, and as mentioned previously, we cannot settle what component from the LFP-motif binding or crystal packing contacts favor the αA1 “OUT” orientation. In KLC1-TPR^[A1-B6]^ (3NF1) and KLC1-TPR^[A1-B5]^ (this study) structures, both with unnatural ligands, the His-tag linker and the αB5’-sym, respectively, are in direct contact with residues from the A1 helix. The His-tag linker in KLC1-TPR^[A1-B6]^ structure (3NF1) makes interactions with the N-terminus of A1 helix which exhibits an “OUT” orientation (Fig 5A). Meanwhile, the αB5’-sym in KLC1-TPR^[A1-B5]^ structure makes interactions with the C-terminus of A1 helix which exhibits an “IN” orientation (Fig 5B). Modeling reveals that the His-tag linker of KLC1-TPR^[A1-B6]^ (3NF1) will generate steric hindrances with αA1 exhibiting a “IN” orientation, in the same way αB5’-sym in the KLC1-TPR^[A1-B5]^ structure will generate steric hindrances with αA1 exhibiting an “OUT” orientation. These latter observations indicate that both His-tag linker and αB5’-sym binding, respectively are only compatible with one orientation of A1 helix. However, based on the structural data available, we cannot conclude if ligand binding recognizes specifically one αA1 orientation or if it induces αA1 reorientation. Altogether, these structural data support that ligand binding into the N-terminal part of the TPR groove can recognize or induce the A1 helix orientation.

### Crystal packing analysis reveals a conserved TPR1:TPR1’ contact

**Fig 6:**
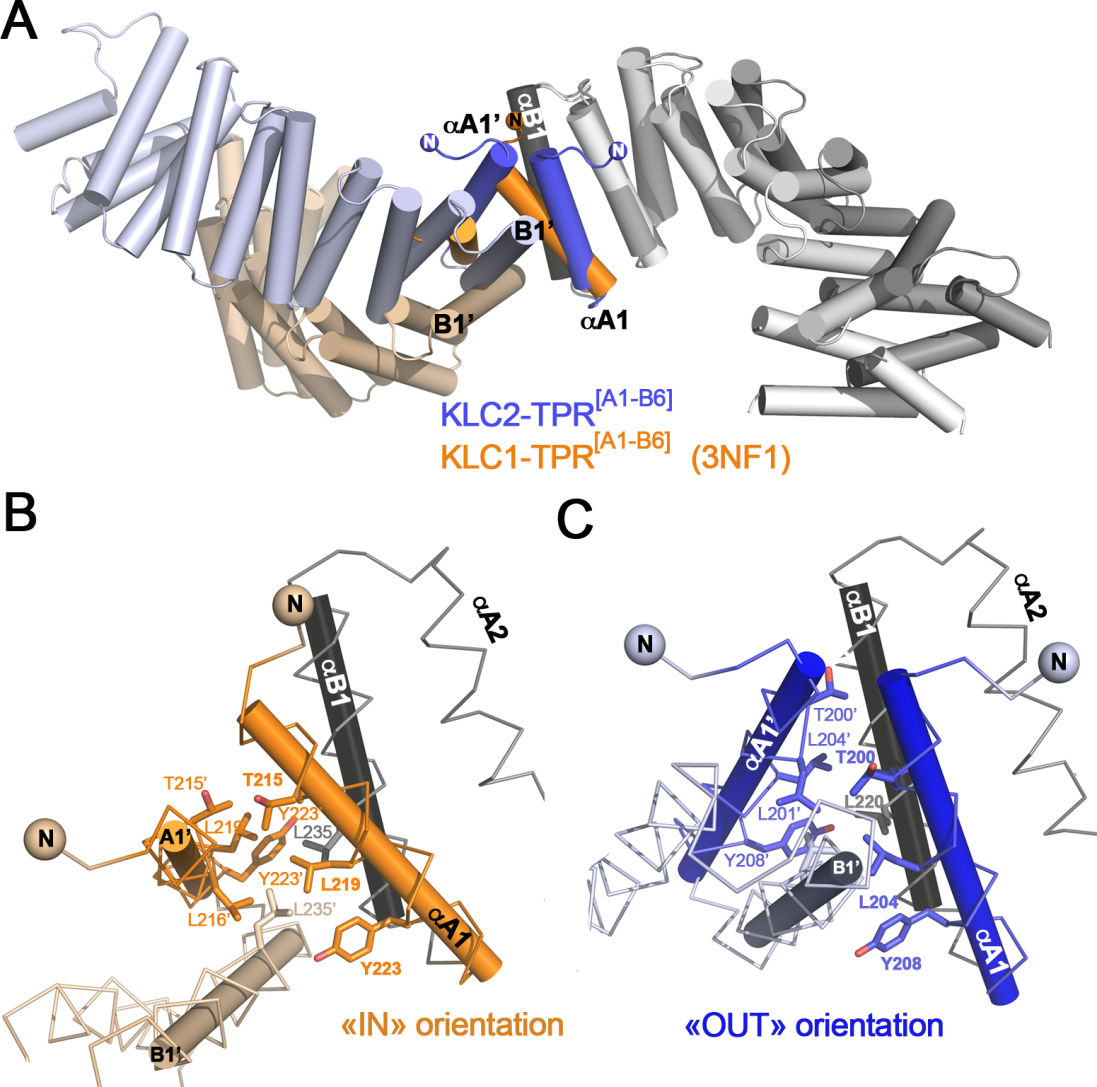
The TPR1:TPR1’ crystal packing contacts. (A) Superposition of KLC2-TPR^[A1-B6]^ (this study, blue) and KLC1-TPR^[A1-B6]^ (3NF1, orange). TPR domain superposition is done on the B1 helix of the main molecule. For clarity, only the TPR domain of KLC2-TPR^[A1-B6]^ (this study) is shown in grey in a cartoon representation as a template, but A1 helices are shown in color. The symmetry molecule at the TPR1:TPR1’ contact is shown in color. A 180° view is shown below. (B) Zoom at the TPR1:TPR1’ contact for the KLC1-TPR^[A1-B6]^ crystal form (3NF1, orange). (C) Zoom at the TPR1:TPR1’ contact for the KLC2-TPR^[A1-B6]^ crystal form (this study, blue). The orientation is conserved between (B) and (C) with the reference B1 helices (grey) indicated with a black axis. Some residues involved in the TPR1:TPR1’ contact are indicated in sticks.

Because A1 helix is the N-terminal capping helix of the KLC-TPR domain, it is not in contact with B_i-1_ helix. Thus, A1 helix is not only exposed at the concave face of the TPR domain, but also at the convex face (Figs 2A and 3A). Taking advantage of the four distinct crystal forms available (Table 2), we analyzed crystal contacts at the N-terminal capping A1 helix. Interestingly, all four crystal forms exhibit crystal contacts at the TPR1 repeat, and all with the TPR1’ repeat of a relative molecule. In the KLC1-TPR^[A1-B5]^ crystal form (this study), only B1 helix is involved in the TPR1:TPR1’ contact, while the “IN” A1 helix is involved in contact with the αB5’-sym that binds into the groove (S4A Fig). Modeling of an “OUT” αA1 orientation in the KLC1-TPR^[A1-B5]^ structure reveals no steric hindrance at the TPR1’ contact, but steric clashes at the αB5’-sym contact. In the three other crystal forms, both A1 and B1 helices are involved in the TPR1:TPR1’ contact. In KLC1-TPR^[A1-B6]^ (3NF1) and KLC2-^LFP^TPR^[A1-B6]^ (5FJY) structures that exhibit an αA1 “OUT” orientation, the TPR1:TPR1’ contacts are virtually identical (S4B Fig), despite being crystallized in different crystal forms (Table 2). Both anti-parallel A1/B1 helices from each molecule lie roughly perpendicular to each other. This arrangement is an adaptation of the TPR1:TPR1’ contact observed in the KLC2-TPR^[A1-B6]^ crystal form (Fig 6A). Main hydrophobic residues involved in the TPR1:TPR1’ contact are conserved and consist of Thr215/200, Leu216/201 and Leu219/204 from the A1 helix and Tyr223/208 and Leu235/220 from B1 helix (KLC1/2 numbering), as well as their relatives from the symmetrical molecule (Fig 6B and 6C). However, the position of these residues relative to each other is slightly different due to their respective αA1 orientations. Thus, in the KLC1-TPR^[A1-B6]^ (3NF1) crystal form, the TPR1:TPR1’ contact buried a total surface area of 1143 Å^2^ which is 25% smaller than that of the KLC2-TPR^[A1-B6]^ structure. Altogether, these four crystal forms reveal that the N-terminal capping A1 helix is a favorable crystal packing binding site whatever its orientation. This highlights the propensity of the A1 helix to be a protein–protein interaction site.

## Conclusions

The TPR domain fold is known to be a versatile module for protein-protein interactions [19]. Previously, the TPR domain of kinesin-light chain (KLC) was shown to undergo an overall closure upon WD-motif cargo binding into the groove, creating a hydrophobic pocket at the αA2/αA3 interface [22]. Such an overall closure movement was also observed with binding of unnatural ligand into the groove (αB5-sym, this study) leading to the formation of the same hydrophobic αA2/αA3 pocket. This overall movement induces different interacting surfaces representing potential determinants for cargo binding and selectivity. Our crystallographic data reveal a local rearrangement at the extreme N-terminal part of the TPR domain. The N-terminal capping A1 helix exhibits structural plasticity adopting two distinct and defined orientations relative to the rest of the TPR domain. Such a difference in orientation causes, at the N-terminal part of the groove, the formation of a hydrophobic pocket at the αA1/αA2 interface, as well as a modification of its surface chemical properties. Based on available structures of KLC1/2-TPR domain containing the N-terminal capping A1 helix, our structural analysis reveals that ligand binding into the groove can be specific of the one or the other orientation. However, understanding if ligand/cargo upon binding into or outside the groove recognizes or induces such an A1 helix rearrangement remains to be carefully studied. Further, our structural analysis reveals that up to now the A1 helix is always involved in crystal packing contacts, especially in a TPR1:TPR1’ contact, which highlights its propensity to be a protein–protein interaction site. In the context of the kinesin1 hetero-tetramer with two KLC molecules being close together, the A1 helix may be responsible for KLC dimerization triggered by dimeric cargo binding, for example with JIP3/4. Altogether, these data underline the structural plasticity of the N-terminal capping A1 helix of the TPR domain of KLC1/2 and invite further questions about the role of this helix in cargo binding and specificity, as well as in putative KLC-TPR dimerization. Further 3D structures of the complete TPR domain of KLC unbound and bound to cargos are needed to investigate these questions and thus better understand the versatility of the TPR domain of KLC for cargo recruitment.

## Supporting information

S1 Fig **SEC-MALLS analysis of KLC2-TPR**^**[A1-B6]**^ **(A) and KLC1-TPR**^**[A1-B5]**^ **(B) fragments.** The size-exclusion profiles of the proteins (monitored by refractometry) and the molecular masses (calculated from light-scattering and refractometry data) are plotted.
(PDF)

S2 Fig Crystal packing at the αB5’-sym contact in KLC1-TPR^[A1-B5]^ structure. **(A)** Details of the interaction between the N-terminal part of the TPR domain (TPR1/2/3 in pink), shown in ribbon, and the C-terminal part of the symmetrical molecule (TPR5 in grey), shown in cartoon. Residues involved in the interface are shown in sticks and hydrogen bonds with dash black lines. **(B)** Surface representation of the αA1/αA2 hydrophobic pocket. Residues forming the pocket are indicated in magenta. The αB5-sym is shown in white ribbon and the Phe416’ that plugs into the pocket, in sticks. **(C)** Superposition of the KLC1-TPR^[A1-B5]^ structure (pink) and the KLC2-TPR^[B1-B5]^:SKIP^WD^ structure (3ZFW; red). Superposition was done on the A2-B2-A3 helices. The αB5’-sym is shown in grey with residues (E415’-S418’) indicated in sticks and the WD-motif from SKIP is shown in red with residues (W207-I212) indicated in sticks.
(PDF)

S3 Fig Natural and unnatural ligands binding to the N-terminal part of the TPR domain groove of KLC. **(A)** Superposition of KLC2-TPR^[B1-B6]^:SKIP^WD^ (3ZFW; red), KLC2-^LFP^TPR^[A1-B6]^ (5FJY, green), KLC1-TPR^[A1-B6]^ (3NF1, orange) and KLC1-TPR^[A1-B5]^ (this study, purple) on the N-terminal part of the TPR domain. The TPR domain of KLC1-TPR^[A1-B6]^ (3NF1) and KLC1-TPR^[A1-B5]^ (this study) are shown in orange light and pink light, respectively with a cartoon/surface representation. The natural and unnatural ligands are shown in cartoon and colored. **(B)** Zoom of the binding interaction of natural and unnatural ligands on the N-terminal part of the TPR domain. Two orthogonal views are shown. Residues indicated in sticks are: Phe202* from the N-terminal Tag sequence in KLC1-TPR^[A1-B6]^ (3NF1, orange), buried in the αA1/αA2 pocket; Trp207 and Leu205 from the WD-motif of SKIP bound to KLC2-TPR^[B1-B6]^ (3ZFW, red), the Trp207 is buried in the αA2/αA3 and the Leu205 is buried in the αA3/αA4 pocket; Phe416’ from the symmetry related molecule bound to KLC1-TPR^[A1-B5]^ (this study, purple), buried in the αA2/αA3 pocket.
(PDF)

S4 Fig The TPR1:TPR1’ crystal packing contacts. (A) KLC1-TPR^[A1-B5]^ crystal form (this study, pink). The second αA1:αB5’ crystal packing contacts are shown in grey. (B) Superposition of KLC1-TPR^[A1-B6]^ (3NF1, orange) and KLC2-^LFP^TPR^[A1-B6]^ (5FYJ, green). TPR domain superposition is done on the B1 helix of the main molecule. A1 helices from the main and the symmetry molecules are shown in dark color.
(PDF)
