# Correction: Structural plasticity of the N-terminal capping helix of the TPR domain of kinesin light chain

**DOI:** 10.1371/journal.pone.0197193

**Published:** 2018-05-07

**Authors:** The Quyen Nguyen, Mélanie Chenon, Fernando Vilela, Christophe Velours, Magali Aumont-Nicaise, Jessica Andreani, Paloma F. Varela, Paola Llinas, Julie Ménétrey

The following information is missing from the Funding section: This work has been supported by iNEXT, grant number 653706, funded by the Horizon 2020 programme of the European Union.
