# Supplementary figures and images for: Structural plasticity of the N-terminal capping helix of the TPR domain of kinesin light chain
Source: PLoS One. 2017 Oct 16;12(10):e0186354. doi: 10.1371/journal.pone.0186354 (PMC5642895; doi:10.1371/journal.pone.0186354)

**A**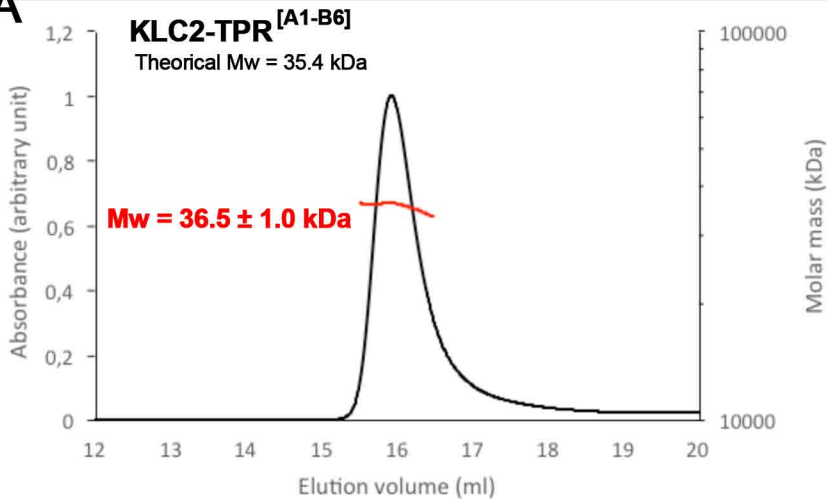**B**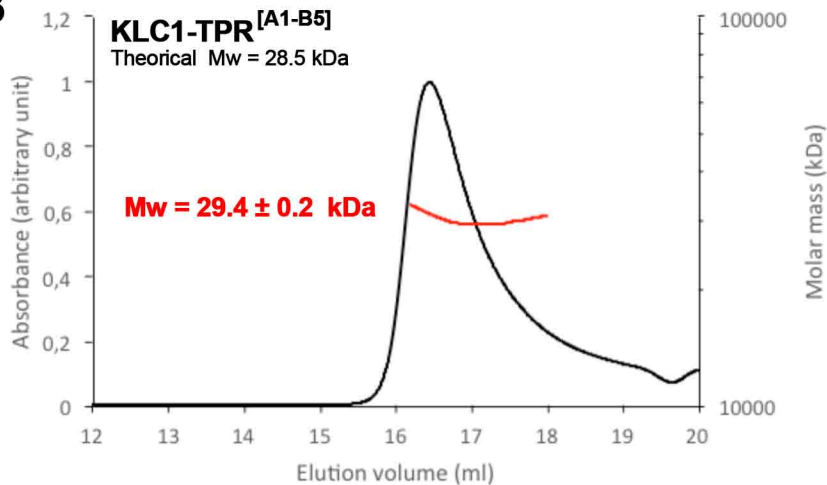

Supplement: S1 Fig — SEC-MALLS analysis of KLC2-TPR[A1-B6] (A) and KLC1-TPR[A1-B5] (B) fragments. The size-exclusion profiles of the proteins (monitored by refractometry) and the molecular masses (calculated from light-scattering and refractometry data) are plotted. (PDF) [file pone.0186354.s001.pdf]

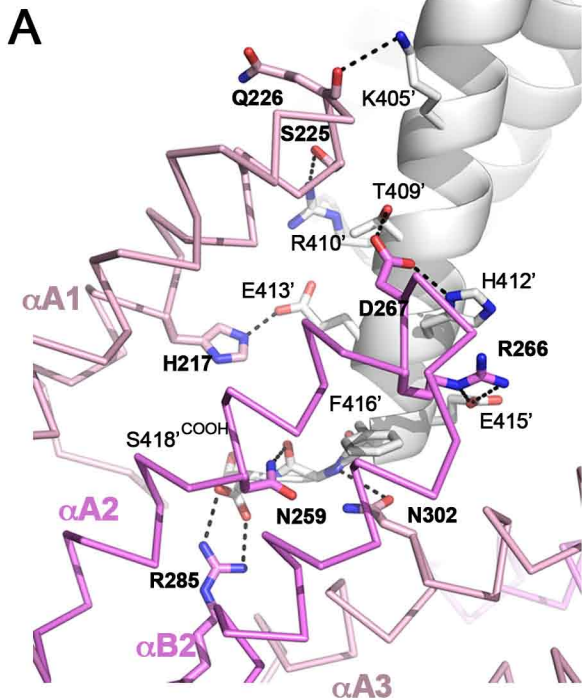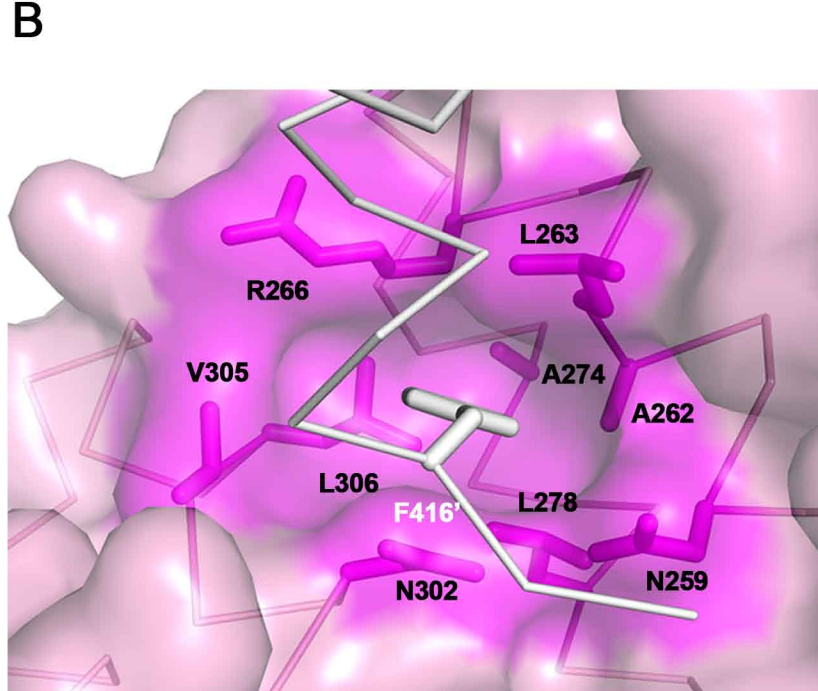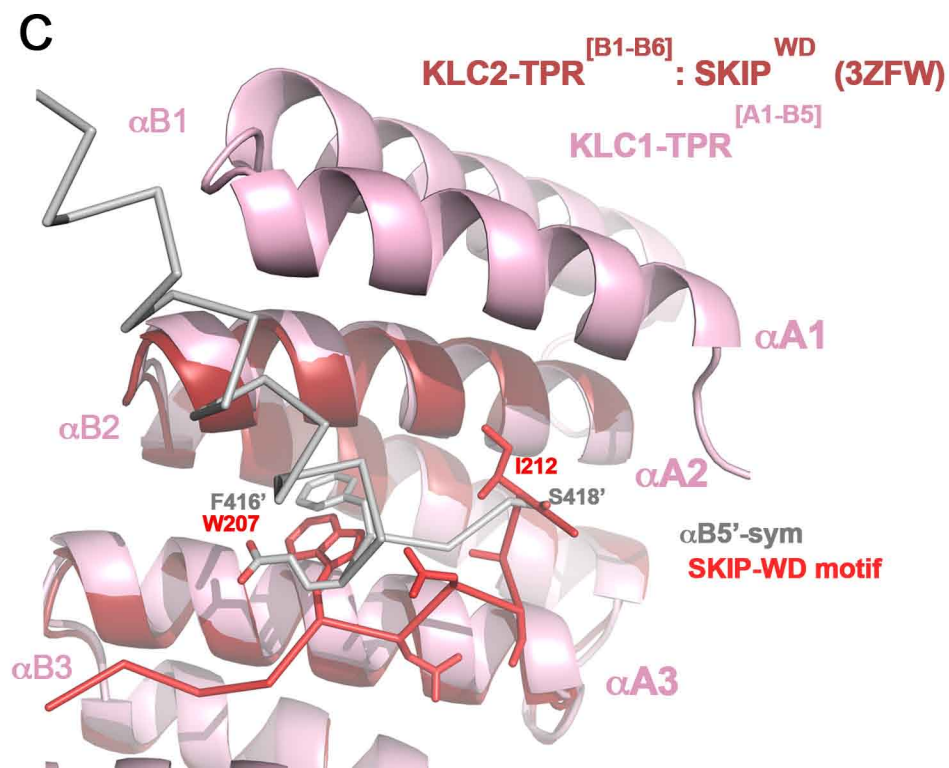

Supplement: S2 Fig — (A) Details of the interaction between the N-terminal part of the TPR domain (TPR1/2/3 in pink), shown in ribbon, and the C-terminal part of the symmetrical molecule (TPR5 in grey), shown in cartoon. Residues involved in the interface are shown in sticks and hydrogen bonds with dash black lines. (B) Surface representation of the αA1/αA2 hydrophobic pocket. Residues forming the pocket are indicated in magenta. The αB5-sym is shown in white ribbon and the Phe416’ that plugs into the pocket, in sticks. (C) Superposition of the KLC1-TPR[A1-B5] structure (pink) and the KLC2-TPR[B1-B5]:SKIPWD structure (3ZFW; red). Superposition was done on the A2-B2-A3 helices. The αB5’-sym is shown in grey with residues (E415’-S418’) indicated in sticks and the WD-motif from SKIP is shown in red with residues (W207-I212) indicated in sticks. (PDF) [file pone.0186354.s002.pdf]

**A****N-terminal part (TPR1/2/3/4)**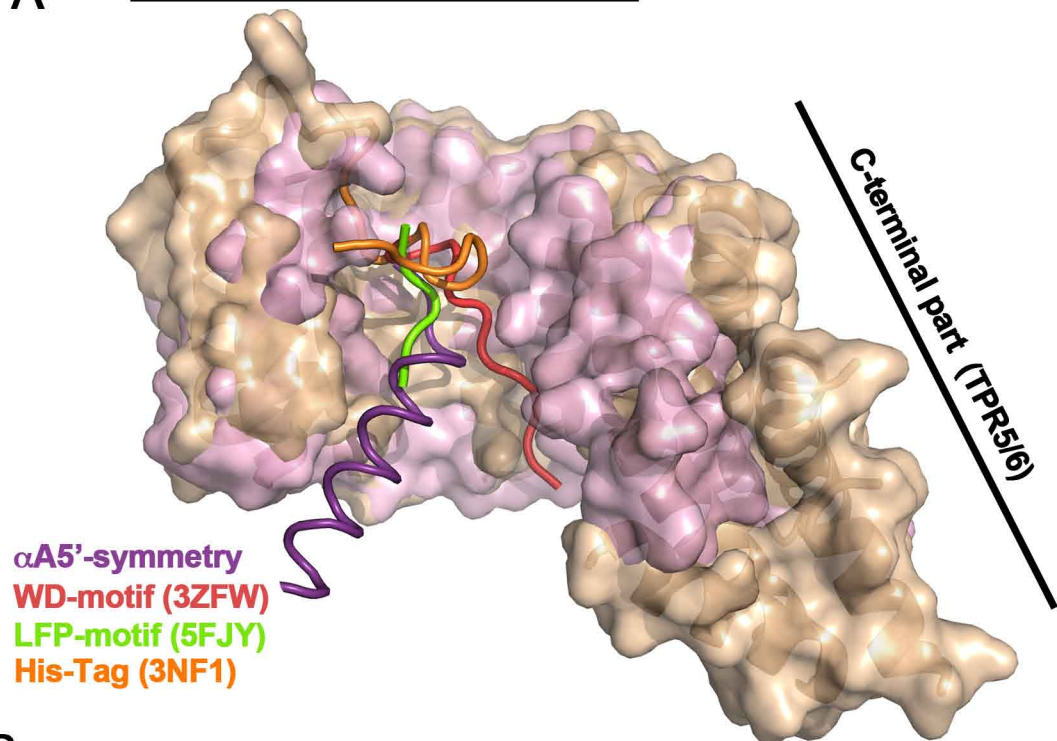

αA5'-symmetry  
WD-motif (3ZFW)  
LFP-motif (5FJY)  
His-Tag (3NF1)

**C-terminal part (TPR5/6)**

**B**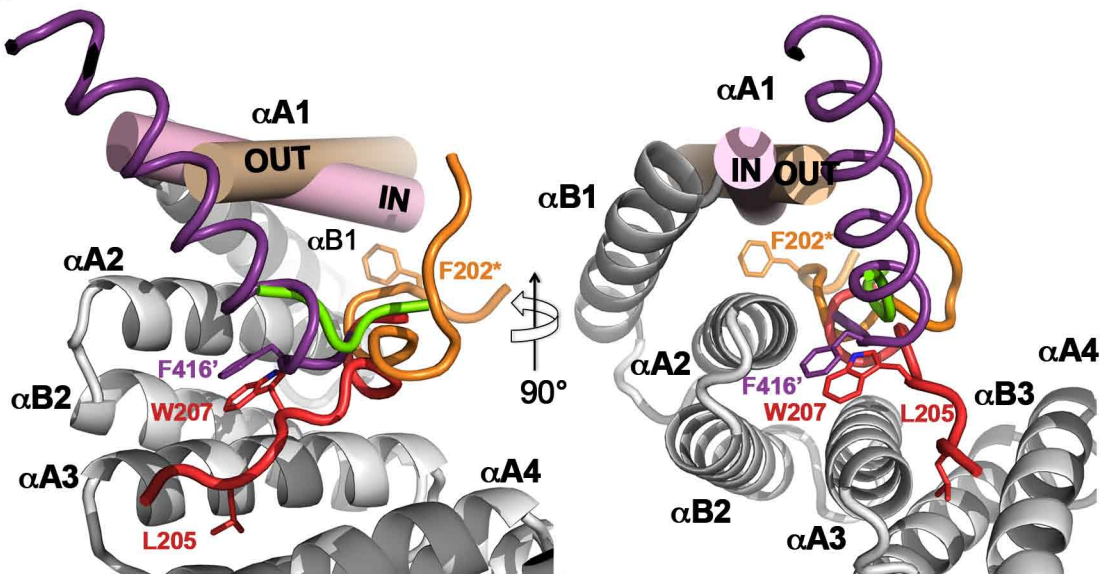

Supplement: S3 Fig — (A) Superposition of KLC2-TPR[B1-B6]:SKIPWD (3ZFW; red), KLC2-LFPTPR[A1-B6] (5FJY, green), KLC1-TPR[A1-B6] (3NF1, orange) and KLC1-TPR[A1-B5] (this study, purple) on the N-terminal part of the TPR domain. The TPR domain of KLC1-TPR[A1-B6] (3NF1) and KLC1-TPR[A1-B5] (this study) are shown in orange light and pink light, respectively with a cartoon/surface representation. The natural and unnatural ligands are shown in cartoon and colored. (B) Zoom of the binding interaction of natural and unnatural ligands on the N-terminal part of the TPR domain. Two orthogonal views are shown. Residues indicated in sticks are: Phe202* from the N-terminal Tag sequence in KLC1-TPR[A1-B6] (3NF1, orange), buried in the αA1/αA2 pocket; Trp207 and Leu205 from the WD-motif of SKIP bound to KLC2-TPR[B1-B6] (3ZFW, red), the Trp207 is buried in the αA2/αA3 and the Leu205 is buried in the αA3/αA4 pocket; Phe416’ from the symmetry related molecule bound to KLC1-TPR[A1-B5] (this study, purple), buried in the αA2/αA3 pocket. (PDF) [file pone.0186354.s003.pdf]

A

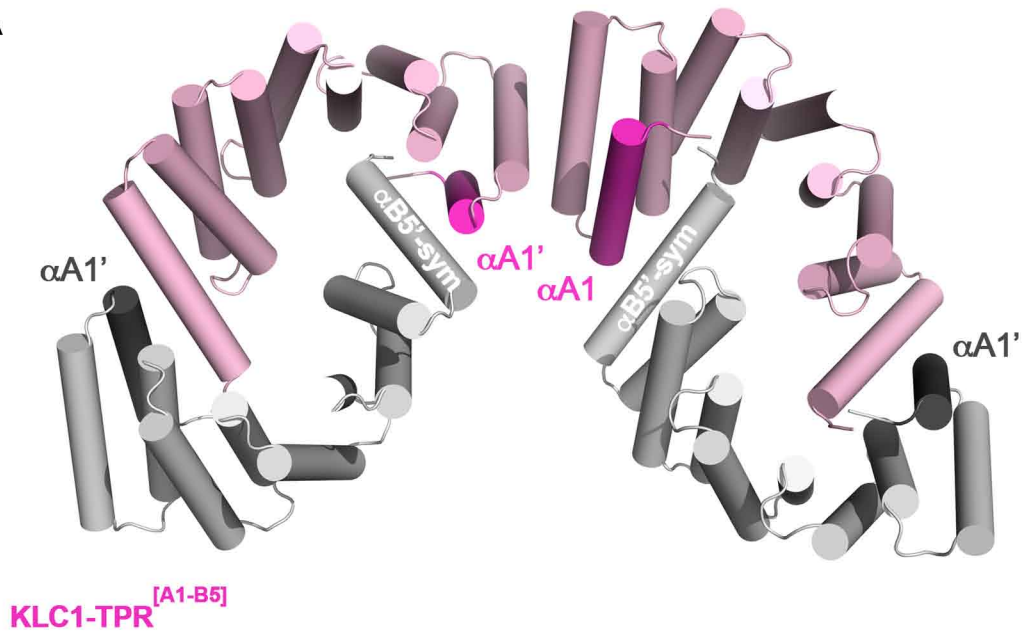

B

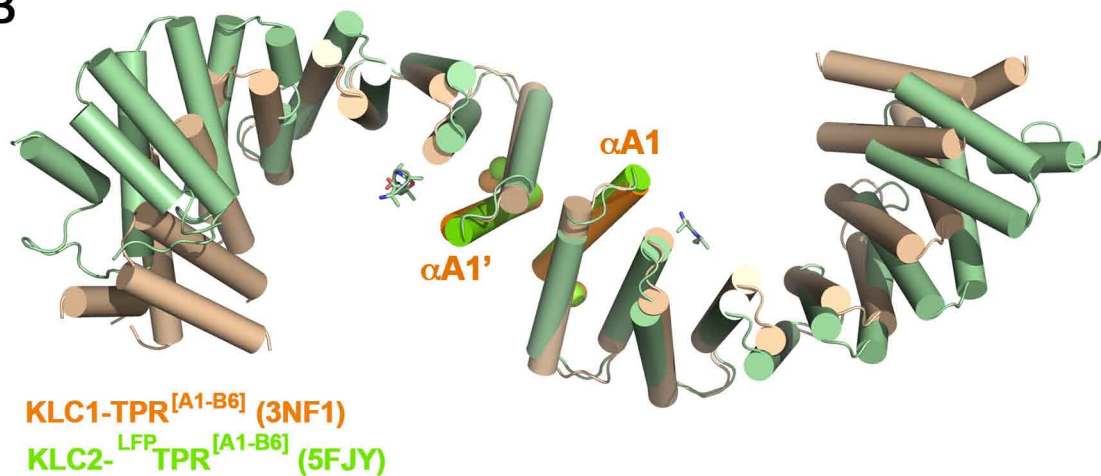

Supplement: S4 Fig — (A) KLC1-TPR[A1-B5] crystal form (this study, pink). The second αA1:αB5’ crystal packing contacts are shown in grey. (B) Superposition of KLC1-TPR[A1-B6] (3NF1, orange) and KLC2-LFPTPR[A1-B6] (5FYJ, green). TPR domain superposition is done on the B1 helix of the main molecule. A1 helices from the main and the symmetry molecules are shown in dark color. (PDF) [file pone.0186354.s004.pdf]
